# Application of α-Syn Real-Time Quaking-Induced Conversion for Brain and Skin Specimens of the Chinese Patients With Parkinson’s Disease

**DOI:** 10.3389/fnagi.2022.898516

**Published:** 2022-07-01

**Authors:** Dong-Dong Chen, Ling Jiao, Yue Huang, Kang Xiao, Li-Ping Gao, Cao Chen, Qi Shi, Xiao-Ping Dong

**Affiliations:** ^1^State Key Laboratory for Infectious Disease Prevention and Control, Collaborative Innovation Center for Diagnosis and Treatment of Infectious Diseases, National Institute for Viral Disease Control and Prevention, Chinese Center for Disease Control and Prevention, Zhejiang University, Beijing, China; ^2^Department of Neurology, The Affiliated Hospital of Guizhou Medical University, Guiyang, China; ^3^Department of Neurology, China National Clinical Research Center for Neurological Diseases, Beijing Tiantan Hospital, Capital Medical University, Beijing, China; ^4^Center for Biosafety Mega-Science, Chinese Academy of Sciences, Wuhan, China; ^5^China Academy of Chinese Medical Sciences, Beijing, China; ^6^Shanghai Institute of Infectious Disease and Biosafety, Shanghai, China

**Keywords:** RT-QuIC, Parkinson’s disease (PD), prion disease (PrD), α-synuclein, PrPsc

## Abstract

The real-time quaking-induced conversion (RT-QuIC) assay has been developed and used as an *in vitro* diagnostic tool for Parkinson’s disease (PD). In this study, we established α-Syn RT-QuIC using recombinant human α-Syn as the substrate. All 5 brain homogenates of neuropathological PD cases and 13 skin homogenates of clinical PD cases showed positive results, whereas all the samples of negative controls remain negative. Meantime, randomly selected 6 skin samples of PD cases and 6 skin samples of sCJD cases showed negative in opposite prion RT-QuIC and α-Syn RT-QuIC. Our α-Syn RT-QuIC showed dose-dependent manner between the lag times and peak ThT fluorescent values. Additionally, the detecting limitation was about 10^–7^ dilution for brain tissues and 10^–6^ for skins. Those data indicate a reliable specificity and good sensitivity of the established α-Syn RT-QuIC in identifying and amplifying the misfolded α-Syn in brain and skin tissues of patients with PD.

## Introduction

Chronic neurodegenerative diseases are a group of neurological diseases due to loss of neurons and/or their myelin sheaths, usually affecting elder people with long clinical course but irreversible disease progression. Neurodegenerative diseases include Alzheimer’s disease (AD), Parkinson’s disease (PD), Huntington disease (HD), amyotrophic lateral sclerosis (ALS), and prion disease (PrD), etc. Although the etiologies of various neurodegenerative diseases are different, aggregates of amyloidogenic proteins in the lesions of central nerve system (CNS) are the common neuropathological features, which are considered as the valuable disease-specific diagnostic biomarkers (Ross and Poirier, 2004; Blennow and Zetterberg, 2018; Steinacker et al., 2019; Compta and Revesz, 2021).

The process of conformational conversion and aggregation of α-Syn in PD is thought like that of PrP^Sc^ in PrD, undergoing prion-like pathology (Mehra et al., 2019; Cazzaniga et al., 2020). Traditionally, the diagnosis of PD is mostly based on the clinical signs, and the definitely confirmed PD relies only upon the identification of substantia nigral α-Syn post-mortem (Dickson, 2018). Immunoassays for α-Syn, both total and phosphorylated, in cerebrospinal fluid (CSF) and other peripheral tissues of PD and DLB have been widely evaluated for diagnostic purpose, but with wide variations (Gelpi et al., 2014; Donadio et al., 2017a; Eusebi et al., 2017; Kalia, 2019). More recently, a real-time quaking-induced conversion (RT-QuIC) using recombinant α-Syn as substrate has been developed and applied in detecting miniscule quantities of abnormal α-Syn in various tissues and body fluids of α-synucleinopathy, particularly in skin specimen, revealing a promising diagnostic value (Fairfoul et al., 2016; Bargar et al., 2021; Donadio et al., 2021; Kuzkina et al., 2021; Mammana et al., 2021; Rossi et al., 2021).

In this study, the α-Syn RT-QuIC assay was established and validated with brain and skin samples of Chinese patients with PD. The specificity and sensitivity of the established α-Syn RT-QuIC were also evaluated with the brain and skin specimens of the patients of other neurological diseases, e.g., neuroglioma, sporadic Creutzfeldt–Jakob disease (sCJD), and other genetic prion diseases. The reactivities of skin samples of patients with PD and sCJD were also cross-evaluated with opposite prion RT-QuIC and α-Syn RT-QuIC. Our results reveal reliable specificity and good sensitivity of the α-Syn RT-QuIC in distinguishing patients with PD and sCJD experimentally.

## Materials and Methods

### Patient Samples

The post-mortem brain samples from the 4 patients of neuropathologically confirmed PD and one of the patients had corresponding skin samples were kindly supplied by the National Prion Disease Pathology Surveillance Center (NPDPSC), Case Western Reserve University School of Medicine, Cleveland, OH, United States. The skin biopsy samples from 12 Chinese patients with PD were obtained from the Department of Neurology, The First Affinitive Hospital, Guizhou Medical University. A number of one post-mortem brain sample and the biopsy skin sample from a patient with neuropathologically confirmed PD were obtained from the Department of Neurology, Beijing Tiantan Hospital, Capital Medical University. Some clinical information of the enrolled patients with PD is shown in Table 1. Patients underwent a detailed clinical research assessment based on the Movement Disorder Society Clinical Diagnostic Criteria for PD (Postuma et al., 2015). Exclusion criteria included oral anticoagulant use, wound healing disorders, or allergic reactions to local anesthetics. Participants were selected during their diagnostic workup or therapy. All patient samples were obtained in accordance with the regulations of the relevant local institutions, and patients and their family members obtained written consent. All samples were stored in the tissue bank of the Center of Chinese CJD Surveillance System, China CDC.

**TABLE 1:** Clinical and demographic data of the recruited patients with PD.

PD cases		Gender	Age (Year)	Disease duration (Year)	Post-mortem (Y/N)
Skin samples	Case 1	Female	57	10	N
	Case 2	Male	54	7	N
	Case 3	Female	75	9	N
	Case 4	Female	66	11	N
	Case 5	Female	62	12	N
	Case 6	Male	57	10	N
	Case 7	Female	67	13	N
	Case 8	Female	68	8	N
	Case 9	Male	57	12	N
	Case 10	Male	64	11	N
	Case 11	Male	74	9	N
	Case 12	Male	67	12	N
	Case 13	Female	68	7	N

### Brain and Skin Homogenate Preparation

Brain homogenates (BH, 10% w/v) were prepared by homogenizing the cortical brain tissues in lysis buffer (10 mM Tris, 10 mM EDTA, 0.5 M EDTA, 10 mM NaCl, 0.5% NP-40, 0.5% sodium deoxycholate) with 0.5 mm zirconium oxide beads with shaking for 1 min at maximum speed. According to the previously described protocols (Donadio et al., 2018; Donadio, 2019), roughly 3 mm × 3-mm to 5 mm × 5-mm punch scalp biopsy (approximately 30–100 mg each in weight) including epidermis and dermis layers was taken by skin sampler at the edge of the left retroauricular incision. Skin samples were immediately fixed in cold Zamboni fixative and kept at 4°C overnight. Skin tissues were cut into small pieces after washed in 1 × Tris-buffered saline (TBS) for three times. Skin homogenates (10% w/v) were prepared according to the protocol described elsewhere (Xiao et al., 2021). Briefly, the skin pieces were incubated with lysis buffer [TBST containing 2 mmol of calcium chloride and 0.25% (w/v) collagenase A] in a constant temperature water bath at 37°C for 4 h. About 0.5-mm zirconium oxide beads were added and shaken at maximum speed for 1 min. After centrifuged at 2,000 *g* for 10 min, the supernatants were collected and stored at –80°C for further usages.

### Real-Time Quaking-Induced Conversion Protocol

Prion RT-QuIC assays were conducted as described previously (Xiao et al., 2018, 2019; Wang et al., 2019). α-Syn RT-QuIC reactions were performed in black 96-well plates with a clear bottom (Nalgene Nunc International) according to the published documents with slight modification (Donadio et al., 2021; Mammana et al., 2021). Recombinant αSyn (r-αSyn) was purchased commercially (rPeptide). A total of 6 glass beads (1 mm in diameter, BioSpec Products) were preloaded in per well. About 2 μl of 10% brain or skin homogenates was mixed with 98 μl of the reaction solution containing 0.1 mg/ml r-αSyn, 40 mM phosphate buffer (pH 8.0), 10 μM ThT, 170 mM NaCl, and 0.00125% SDS in final concentration. After sealed with a clear sealing film (ThermoFisher), the plate was placed into BMG FLUOstar Omega plate reader. The working condition was temperature, 42°C; vibration speed, 400 rpm double orbital; vibration/incubation time, 60/60 s; reaction time, 80–140. ThT fluorescence (excitation wavelength, 450 nm; emission wavelength, 480 nm) each reaction was automatically measured every 45 min and expressed as relative fluorescence unit (rfu). The cutoff value was set as the mean value of the negative controls plus 10 times the standard deviation. A sample was considered as positive when ≥ 2 wells out of 3 and 4 wells showed positive reaction curves. RT-QuIC was repeated for the sample with only 1 well positive.

### Statistical Analysis

The lag times and the peak fluorescence values were analyzed and plotted by the software GraphPad Prism.

## Results

### Validation of α-Syn Real-Time Quaking-Induced Conversion Assay With the Brain Samples of Neuropathologically Confirmed Parkinson’s Disease Cases

**FIGURE 1:**
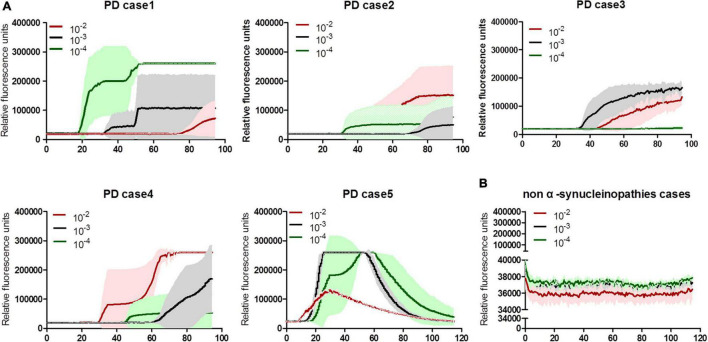
α-Syn RT-QuIC spectra of serially diluted brain homogenates of neuropathological confirmed PD and non-α-synucleinopathy cases. **(A)** Individual 5 cases of PD. **(B)** Summarized spectra non-α-synucleinopathy cases, including a normal person, 8 cases of neuroglioma, 3 cases of FFI, and 1 G114V gCJD. The dilutions are indicated on the top. *Y*-axis represents rfu (ThT value). *X*-axis represents the time (h) post-reaction. Each reaction consists of 3–4 duplicates.

To validate the specificity and sensitivity of the established α-Syn RT-QuIC assay, the stored brain samples from 5 neuropathologically confirmed patients with PD, together with those from a healthy person died in a car accident, 8 cases of neuroglioma, 3 cases of fatal familial insomnia (FFI), and 1 G114V genetic CJD (gCJD), were recruited into this study. After prepared brain homogenates, 10^–2^, 10^–3^, and 10^–4^ diluted preparations of each sample were prepared, respectively. All 5 samples from PD cases were positive in α-Syn RT-QuIC (Figure 1A). Further analysis of the reactivity in different dilutions revealed all (5/5) positive in 10^–2^ dilution, 4/5 positive in 10^–3^, and 3/5 positive in 10^–4^ dilution (Table 2). Contrarily, all 13 brain samples of non-α-synucleinopathies cases, including a normal person, 8 neuroglioma cases, 3 FFI cases, and 1 G114V gCJD case, were negative in α-Syn RT-QuIC (Figure 1B and Table 2). Notably, the sample from a patient with glioma showed a weak positive reaction in one well of three repeats at 10^–3^ dilution, in which the fluorescence value was higher but close to the threshold value. Repeating tests of the sample from this case did not elicit the positive reaction in all three wells. According to the judgment criteria, this case was considered as negative. Those data indicate that positive α-Syn RT-QuIC reactions can be elicited by the brain tissues of patients with PD with reliable specificity.

**TABLE 2:** The results of the brain samples in per well at the different dilutions from patients with PD and non-α-synucleinopathies cases.

Diagnosis	No.	Dilution			Final result
			
		10^–2^	10^–3^	10^–4^	
PD	Case 1	_+/+/–_	_+/+/–_	_+/+/+/+_	_+_
	Case 2	_+/+/_	_±/–/–_	_+/+/–_	_+_
	Case 3	_+/+/+/+_	_+/+/+/+_	_–/–/–/–_	_+_
	Case 4	_+/+/+/+_	_+/+/±_	_±/–/–_	_+_
	Case 5	_+/+/+_	_+/+/+_	_+/+/+_	_+_
Healthy	Case 1	_–/–/–_	_–/–/–_	_–/–/–_	–
Glioma	Case 1	_–/–/+_	_–/–/–_	_–/–/–_	–
	Case 2	_–/–/–_	_–/–/–_	_–/–/–_	–
	Case 3	_–/–/–_	_–/–/–_	_–/–/–_	–
	Case 4	_–/–/–_	_–/–/–_	_–/–/–_	–
	Case 5	_–/–/–_	_–/–/–_	_–/–/–_	–
	Case 6	_–/–/–_	_–/–/–_	_–/–/–_	–
	Case 7	_–/–/–_	_–/–/–_	_–/–/–_	–
	Case 8	_–/–/–_	_–/–/–_	_–/–/–_	–
FFI	Case 1	_–/–/–_	_–/–/–_	_–/–/–_	–
	Case 2	_–/–/–_	_–/–/–_	_–/–/–_	–
	Case 3	_–/–/–_	_–/–/–_	_–/–/–_	–
G114V gCJD	Case 1	_–/–/–_	_–/–/–_	_–/–/–_	–

### Validation of α-Syn Real-Time Quaking-Induced Conversion With the Biopsy Skin Samples of Parkinson’s Disease and Sporadic CJD Cases

**FIGURE 2:**
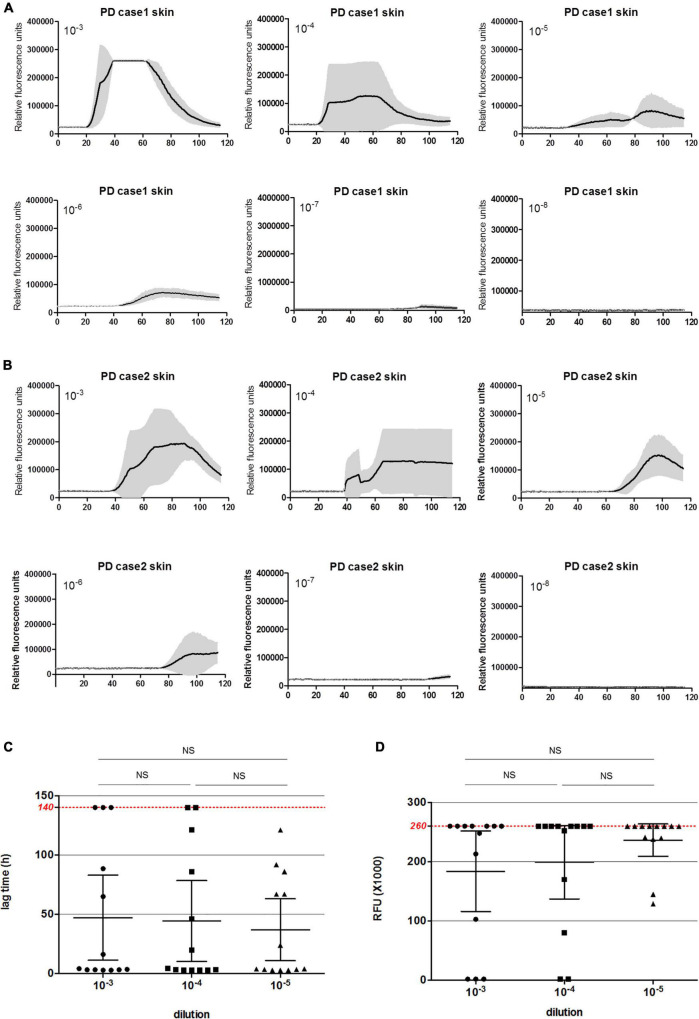
α-Syn RT-QuIC of serially diluted skin homogenates of 13 clinical diagnosed patients with PD and 15 sCJD cases. **(A)** α-Syn RT-QuIC spectra of individual patients with PD. *Y*-axis represents rfu (ThT value). *X*-axis represents the time (h) post-reaction. Each reaction consists of 3 duplicates. **(B)** Summarized α-Syn RT-QuIC spectra of patients with sCJD. Each reaction consisted of 3 duplicates. **(C)** The lag times of three dilutions of the skin specimens of 13 PD cases. Maximal reaction time (140 h) is indicated with red dot line in the graph. **(D)** The peak rfu values of three dilutions of the skin specimens of 13 PD cases. Maximal detecting limitation of ThT value is indicated with red dot line in the graph. Ns means there is no statistical difference.

To evaluate the potential of the established α-Syn RT-QuIC in the usage for biopsy skin samples of patients with PD, the biopsy skin samples from 13 clinically diagnosed patients with PD and 15 patients with probable sCJD were collected. The sampling sites of all tested skin specimens of patients with PD were scalp, whereas that of sCJD cases were various, including from head, trunk, and limbs (Table 3). All 15 skin samples of sCJD cases were verified to be positive in prion RT-QuIC previously. There were no significant differences in detection results between samples from different parts of the sCJD cases (Xiao et al., 2021). About 10^–3^, 10^–4^, and 10^–5^ diluted skin homogenates were separately prepared. Positive α-Syn RT-QuIC was considered in all skin samples of 13 PD cases (Figure 2A and Table 3). All 15 sCJD cases still maintained negative after 140 h of reaction (Figure 2B and Table 3). Calculation of the positive numbers in different dilutions showed 10 in 10^–3^, 11 in 10^–4^, and 13 in 10^–5^ (Table 3). A total of seven skin specimens displayed the similar RT-QuIC reactive curves in all three dilutions, which started to increase in 3–5 h post-reaction, reached to detecting limitation of ThT value (rfu 260,000) rapidly, and dropped down in some wells 80–120 h afterward. Others had relatively long lag times and low ThT peaks. A total of two cases (Cases 5 and 7) showed negative in 10^–3^ and 10^–4^ dilutions but positive in 10^–5^ dilution, whereas one case (Case 11) was negative in 10^–3^ but positive in 10^–4^ and 10^–5^. Repeat α-Syn RT-QuIC tests of these three cases illustrated the same results (Figure 2A).

**TABLE 3:** The results of the skin samples in per well at the different dilutions from patients with PD and sCJD.

Diagnosis	No.	Sampling site	Dilution			Final result
				
			10^–3^	10^–4^	10^–5^	
PD	Case 1	scalp	_+/+/+_	_+/+/+_	_+/+/+_	_+_
	Case 2	scalp	_+/+/+_	_+/+/+_	_+/+/+_	_+_
	Case 3	scalp	_+/+/+_	_+/+/+_	_+/+/+_	_+_
	Case 4	scalp	_+/+/+_	_+/+/+_	_+/+/+_	_+_
	Case 5	scalp	_–/–/–_	_–/–/–_	_–/+/+_	_+_
	Case 6	scalp	_+/±_	_+/+/+_	_+/+/+_	_+_
	Case 7	scalp	_–/–/–_	_±/–_	_+/+/+_	_+_
	Case 8	scalp	_+/+/+_	_+/+/+_	_+/+/+_	_+_
	Case 9	scalp	_+/+/+_	_+/+/+_	_+/+/+_	_+_
	Case 10	scalp	_+/+/+_	_+/+/+_	_+/+/+_	_+_
	Case 11	scalp	_–/–/–_	_+/+/+_	_+/+/+_	_+_
	Case 12	scalp	_+/+/+_	_+/+/+_	_+/+/+_	_+_
	Case 13	scalp	_+/+/+_	_+/+/+_	_+/+/+_	_+_
sCJD	Case 1	abdomen	_–/–/–_	_–/–/–_	_–/–/–_	_–_
	Case 2	lateral upper arm	_–/–/–_	_–/–/–_	_–/–/–_	_–_
	Case 3	neck shoulder junction	_–/–/–_	_–/–/–_	_–/–/–_	_–_
	Case 4	medial upper arm	_–/–/–_	_–/–/–_	_–/–/–_	_–_
	Case 5	medial upper arm	_–/–/–_	_–/–/–_	_–/–/–_	_–_
	Case 6	Inner thighs	_–/–/–_	_–/–/–_	_–/–/–_	_–_
	Case 7	posterior neck	_–/–/–_	_–/–/–_	_–/–/–_	_–_
	Case 8	medial upper arm	_–/–/–_	_–/–/–_	_–/–/–_	_–_
	Case 9	behind ear	_–/–/–_	_–/–/–_	_–/–/–_	_–_
	Case 10	abdomen	_–/–/–_	_–/–/–_	_–/–/–_	_–_
	Case 11	neck	_–/–/–_	_–/–/–_	_–/–/–_	_–_
	Case 12	medial upper arm	_–/–/–_	_–/–/–_	_–/–/–_	_–_
	Case 13	neck	_–/–/–_	_–/–/–_	_–/–/–_	_–_
	Case 14	medial upper arm	_–/–/–_	_–/–/–_	_–/–/–_	_–_
	Case 15	abdomen	_–/–/–_	_–/–/–_	_–/–/–_	_–_

The average lag times of positive conversion and the peak values of RFU of 13 tested samples in each dilution were individually calculated. For lag time (Figure 2C), among 10 positive samples in the dilution of 10^–3^, 8 were less than 50 h, and 2 were in the range of 51–100 h. Among 11 positive reactions in the dilution of 10^–4^, 9 were less than 50 h, 1 was in the range of 51–100 h, and 1 was long than 100 h post-reaction. Among 13 positive samples in the dilution of 10^–5^, 8 were less than 50 h, 4 was in the range of 51–100 h, and 1 was long than 100 h post-reaction. The average lag time of 10^–5^ preparation was slightly shorter than that of 10^–4^ and 10^–3^ dilutions, but without statistical difference. For peaks of rfu (Figure 2D), 9 were over 200,000 rfu, 1 was close to 100,000 rfu in the group of 10^–3^, 9 were over 200,000 rfu, 1 was in the range of 50,000–100,000 rfu, and 1 was less than 50,000 rfu in that of 10^–4^, whereas 11 were over 200,000 rfu and 2 were in the range of 50,000–100,000 rfu. Although the average peak rfu value of the positive reactions in the group of 10^–5^ was higher than the group of 10^–4^ and 10^–3^, statistical analysis of rfu values did not reveal the difference between three groups.

### Evaluation of the Reactivities of the Skin Samples of Parkinson’s Disease and Sporadic CJD Cases in the Opposite Real-Time Quaking-Induced Conversion Assays

To further evaluate the specificities of the established α-Syn RT-QuIC and prion RT-QuIC methods, the skin samples of six patients with PD showing positive in α-Syn RT-QuIC were randomly selected and subjected into prion RT-QuIC assay, whereas those of six sCJD cases showing positive in prion RT-QuIC were employed into α-Syn RT-QuIC test. The tests were conducted with 10^–4^ diluted skin homogenates. Under our experimental condition, none of the six PD samples showed positive in prion RT-QuIC, whereas none of the six sCJD samples produced positive reaction in α-Syn RT-QuIC (Table 4). This result indicates good specificities of the established α-Syn RT-QuIC and prion RT-QuIC in recognizing and amplifying the respective target agents.

**TABLE 4:** The reactivities of the skin samples (10^–4^ diluted) from six PD cases and six sCJD cases in α-syn RT-QuIC and prion RT-QuIC assays.

Diagnosis	No.	α-syn RT-QuIC	prion RT-QuIC
PD	Case 1	+	–
	Case 2	+	–
	Case 3	+	–
	Case 4	+	–
	Case 5	+	–
	Case 6	+	–
sCJD	Case 1	–	+
	Case 2	–	+
	Case 3	–	+
	Case 4	–	+
	Case 5	–	+
	Case 6	–	+

### Evaluation of Detecting Limitation of α-Syn Real-Time Quaking-Induced Conversion in Skin and Brain Samples of Two Parkinson’s Disease Cases

**FIGURE 3:**
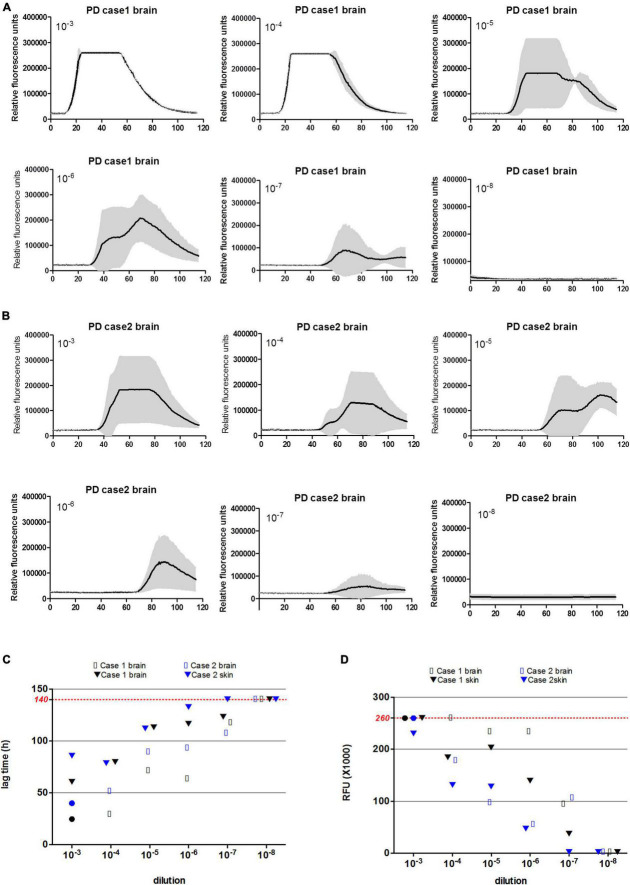
α-Syn RT-QuIC of serially diluted brain and scalp skin homogenates of two neuropathologically confirmed PD cases. **(A)** α-Syn RT-QuIC spectra of Case 1. **(B)** α-Syn RT-QuIC spectra of Case 2. The dilutions are indicated on the top. *Y*-axis represents rfu (ThT value). *X*-axis represents the time (h) post-reaction. Each reaction consists of 3 duplicates. **(C)** The lag times of serially diluted (from 10^–3^ to 10^–8^) paired brain and skin specimens of two PD cases. Maximal reaction time (140 h) is indicated with red dot line in the graph. **(D)** The peak rfu values of serially diluted (from 10^–3^ to 10^–8^) paired brain and skin specimens of two PD cases. Maximal detecting limitation of ThT value is indicated with red dot line in the graph.

To evaluate the detecting limitations of α-Syn RT-QuIC in the skin and brain samples, the brain and skin homogenates from two PD cases were serially diluted from 10^–3^ to 10^–8^ and subjected into α-Syn RT-QuIC with three duplicated wells. Positive reactivities were noted in the dilutions from 10^–3^ to 10^–7^ in skin specimens and in that from 10^–3^ to 10^–6^ in brain ones of Case 1 (Figure 3A), whereas positive reactivities were recorded in the dilutions from 10^–3^ to 10^–6^ in both skin and brain preparations of Case 2 (Figure 3B). Furthermore, the average lag time and the peak rfu value of each sample were calculated separately. Both lag time (Figure 3C) and the peak rfu (Figure 3D) showed the dose-dependent manner that along with the dilutions of the tested samples, the lag times increased and the peak rfu values decreased. The seeding capacities of brain samples in α-Syn RT-QuIC under the current experimental conditions looked slightly stronger than that of skin samples in some preparations, e.g., 10^–3^, 10^–4^, and 10^–6^, whereas those of Case 1 seemed to be stronger than Case 2, but without statistical difference. These results highlight that the detecting limitations of the established α-Syn RT-QuIC in the skin and brain samples of PD cases range between 10^–6^ and 10^–7^ dilutions.

## Discussion

Real-time quaking-induced conversion technique, by inducing conversion of recombinant target proteins *via* intermittent shaking, has been widely evaluated and applied in the diagnosis of some neurodegenerative diseases, such as PrD and PD (Wu et al., 2021). In this study, we have validated the potential of the established α-Syn RT-QuIC on identification and amplification of trace amount of abnormal α-Syn in the brain and skin specimens of patients with PD. Good sensitivity and reliable specificity of the α-Syn RT-QuIC have been achieved based on a testing panel consisting of post-mortem brain samples of 5 PD cases and biopsy skin samples of 13 PD cases, together with dozens of brain and skin specimens of neuroglioma, sCJD, FFI, and gCJD cases.

Although case numbers in this study are relatively limited that makes us hesitant to address the diagnostic accuracy of the established α-Syn RT-QuIC, positive in all 5 brain and 13 skin samples of PD and negative in all controls of other diseases propose a significant potential in the future usage. Higher diagnostic accuracies (over 90%) of α-Syn RT-QuIC for α-synucleinopathy have been reported in some previous studies with relatively large case numbers (Bargar et al., 2021; Donadio et al., 2021; Kuzkina et al., 2021; Mammana et al., 2021). High accuracies of PD diagnosis are obtained not only from skin tissues but also from many other CNS and peripheral tissues, such as brains, CSF, salivary gland, and colon (Bargar et al., 2021). As an easily obtained biopsy specimen, RT-QuIC of skin tissues has already showed more accessibility in the diagnosis of PD and PrD (Wang et al., 2019; Bargar et al., 2021; Wu et al., 2021; Xiao et al., 2021). Moreover, Manne et al. (2020) have reported the usage of α-Syn RT-QuIC with formalin-fixed paraffin-embedded skin sections of PD cases with 75% of sensitivity and 83% of specificity, highlighting a much wide usage.

Some studies of skin prion RT-QuIC have revealed a variation in seeding activity based on the sampling sites. The skin specimens from ears and back of prion infected experimental rodents showed slightly higher seeding activities than that from limbs (Wang et al., 2019). The skins near the ear of sCJD cases also elicited relatively strong seeding activities compared to that from low back and skull apex (Orrú et al., 2017). However, other studies including ours seem not to figure out significant association between skin sampling location and prion seeding activity (Mammana et al., 2020; Xiao et al., 2021). An α-Syn RT-QuIC assay with small case numbers shows limitedly strong seeding activity of the skin sample from cervical area than that of thigh, without significance in the kinetics of positive replicates (Mammana et al., 2021). Another study with the skin samples of cervical, lower back, thigh, and lower leg reveals similar amplifying kinetics in α-Syn RT-QuIC (Kuzkina et al., 2021). Additionally, immunofluorescent assays demonstrate more frequent depositions of misfolded α-Syn in proximate (100% in the cervical C7 site) compared to distal (35% in the thoracic T12 region) skin areas of patients with PD (Donadio et al., 2014, 2017b; Doppler et al., 2014). Whether such difference affects the reactivity of α-Syn RT-QuIC in Chinese patients with α-synucleinopathy needs large number size tests.

It is reasonable to believe that misfolded α-Syn deposits mostly in CNS tissues of patients with PD. Analysis of two PD cases with the paired brain and skin specimens in this study shows slightly stronger but insignificant α-Syn seeding activity of brain samples than skin ones. One study demonstrates that the seeding activity of skin samples (SD_50_/mg: 5 × 10^4^) in α-Syn RT-QuIC is lower than that of brains (SD_50_/mg: 2.8 × 10^6^) (Bargar et al., 2021). However, such difference between brain and skin tissues of patients with PD in seeding activity in α-Syn RT-QuIC seems to be much less compared to that of PrD cases in prion RT-QuIC. In scrapie 263K-infected hamsters, the seeding activity of brains in prion RT-QuIC is about 10^4^-folds higher that of skins, whereas in the sCJDMM1-infected Tg40h mice, the seeding activity of brains is also > 10^4^-folds higher that of skins (Wang et al., 2019). Study on the sCJD also identifies approximately 10^–3^ to 10^–5^-folds lower seeding activity of skin samples compared to that of brains (Orrú et al., 2017). Our studies also demonstrate sufficient RT-QuIC seeding activities of the brains and CSFs of FFI, a rare genetic PrD (gPrD) with D178N/M129M genotype in *PRNP* usually containing much less PrP*^Sc^* in the brain tissues, whereas the skin samples of FFI cases fail to elicit positive reactivity (Xiao et al., 2019, 2020, 2021). Unlike the wide distributions of misfolded α-Syn in skin tissues of patients with PD, PrP*^Sc^* is extremely rarely detectable in skins of the patients with sCJD and gPrD, as well as in the prion-infected rodents, although markedly weak PrP*^Sc^* signal is observable in the skin samples of a sCJD case and a variant CJD (vCJD) in US after undergone a complicate approach of enrichment (Orrú et al., 2017). Such distributing difference of the abnormally misfolded proteins in CNS and other peripheral tissues between PD and PrD may partially explain the difference in their RT-QuIC seeding activities.

Under our experimental condition, higher diluted (10^–5^) skin samples of PD cases seem to induce more positive reactions in α-Syn RT-QuIC than lower diluted (10^–3^ and 10^–4^) ones. Higher concentration of samples but lower capacity in the aggregation of α-Syn may reflect that some unidentified components in the tested samples are not conducive to the nucleation and growth of α-Syn aggregates (Bellomo et al., 2021). Less seeding activity of larger quantity of CSF of sCJD cases is also observed in prion RT-QuIC (Xiao et al., 2018). The presence of unknown inhibitor(s) in the tested samples, particularly in the relatively high concentrated sample, is probably associated with such phenomenon. We have also noticed that some samples display positive reactive curves with long lag phase, even 100 h post-reaction. We recommend repetition of tests for those cases to avoid of false positive. Meanwhile, the diagnostic values of those cases should be carefully evaluated together with the data of clinical and other examinations. Based on our preliminary results, we suggest that skin α-Syn RT-QuIC is conducted in the condition of at least two dilutions (10^–4^ and 10^–5^) with 3–4 duplicating wells and the reaction time of 120 h for future application. The diagnostic significance of the established α-Syn RT-QuIC of biopsy skin specimens for Chinese patients of α-synucleinopathy still needs large-scale clinical trials.

## Data Availability Statement

The original contributions presented in this study are included in the article/supplementary material, further inquiries can be directed to the corresponding authors.

## Ethics Statement

The studies involving human participants were reviewed and approved by the Ethics Committee of the National Institute for Viral Disease Control and Prevention, China CDC, under the protocol of 2009ZX10004-101 and by the Institutional Review Boards (IRB) of the University Hospital Cleveland Medical Center Written informed consent was obtained from family members for skin autopsy through the NPDPSC. The patients/participants provided their written informed consent to participate in this study.

## Author Contributions

D-DC contributed to study design, performed the experiments, and prepared the manuscript. LJ and YH assisted with collecting and organizing clinical cases. KX, L-PG, and CC assisted with the preparation of experimental materials and statistical analysis. QS contributed to the study design and collect clinical cases. X-PD and QS contributed to the design, study concept, and final manuscript preparation. All authors read and approved the final manuscript.

## Conflict of Interest

The authors declare that the research was conducted in the absence of any commercial or financial relationships that could be construed as a potential conflict of interest.

## Publisher’s Note

All claims expressed in this article are solely those of the authors and do not necessarily represent those of their affiliated organizations, or those of the publisher, the editors and the reviewers. Any product that may be evaluated in this article, or claim that may be made by its manufacturer, is not guaranteed or endorsed by the publisher.

## Abbreviations

PD: Parkinson’s disease
α-Syn: α-synuclein
CNS: central nerve system
RT-QuIC: real-time quaking-induced conversion
FFI: fatal familial insomnia
gCJD: genetic Creutzfeldt–Jakob disease
sCJD: sporadic CJD
PrD: prion disease
AD: Alzheimer’s disease
HD: Huntington’s disease
ALS: amyotrophic lateral sclerosis
PrP ^C^: prion protein
PrP ^Sc^: scrapie isoform of PrP
CSF: cerebrospinal fluid
OM: olfactory mucosa
DLB: Lewy bodies
MSA: multiple system atrophy.
